# Cyclin D1 and PRAME expression in distinguishing melanoma in situ from benign melanocytic proliferation of the nail unit

**DOI:** 10.1186/s13000-022-01218-3

**Published:** 2022-04-28

**Authors:** Young Jae Kim, Chang Jin Jung, Hyoungmin Na, Woo Jin Lee, Sung Eun Chang, Mi Woo Lee, Chan-Sik Park, Youngkyoung Lim, Chong Hyun Won

**Affiliations:** 1 Department of Dermatology, Asan Medical Center, University of Ulsan College of Medicine, 88 Olympic- ro 43 gil, Songpa-gu, Seoul, Korea; 2 Department of Pathology, Asan Medical Center, University of Ulsan College of Medicine, 88 Olympic- ro 43 gil, Songpa-gu, Seoul, Korea; 3 Present address: Department of Dermatology, Seoul National University Hospital, 101, Daehak ro, Jongno gu, Seoul, Korea

**Keywords:** Cyclin D1, Melanoma, Nail, PRAME, Retrospective study

## Abstract

**Background:**

Distinguishing benign lesion from early malignancy in melanocytic lesions of the nail unit still remains a diagnostic challenge, both clinically and histopathologically. While several immunohistochemistry (IHC) stainings have been suggested to help discriminate benign subungual melanocytic proliferation (SMP) and subungual melanoma in situ (MIS), the diagnostic utility of IHC staining for cyclin D1 and PRAME has not been thoroughly investigated in melanocytic lesions of nail unit.

**Methods:**

This retrospective study included cases of benign SMP and subungual MIS confirmed by biopsy at Asan Medical Center from January 2016 to December 2020. Cases of melanocytic activation without proliferation and melanoma where dermal invasion was identified were excluded. Cyclin D1 and PRAME expression was assessed by counting proportion of melanocytes with nuclear positivity under 200x magnification.

**Results:**

A total of 14 patients with benign SMP and 13 patients with subungual MIS were included in this study. 11 patients with benign SMP (71.4%) and 5 patients with subungual MIS (38.5%) showed > 60% nuclear immunostaining for cyclin D1, respectively. While 13 patients with benign SMP (92.9%) showed totally negative staining for PRAME, 10 patients with subungual MIS (76.9%) exhibited > 50% nuclear immunostaining for PRAME. Using the cutoff of 10%, PRAME exhibited good overall discrimination between benign SMP and subungual MIS (AUC = 0.849, 95% CI = 0.659–0.957).

**Conclusions:**

This study suggests that PRAME IHC staining as a reliable discriminator in distinguishing subungual MIS from benign SMP.

**Supplementary information:**

The online version contains supplementary material available at 10.1186/s13000-022-01218-3.

## Background

It still remains a diagnostic challenge to distinguish benign lesion from early malignancy in melanocytic lesions of the nail unit, both clinically and histopathologically [1, 2]. Although non-invasive diagnostic tools including the “ABCDEF rule” had been proposed for early clinical detection of subungual melanoma [3], still the biopsy and histopathological evaluation is the gold standard in distinguishing subungual melanoma in situ (MIS) from benign subungual melanocytic proliferation (SMP) including lentigo and melanocytic nevus [4]. Especially, assessing melanocyte density along the dermoepidermal junction of nail unit has been proposed to be a reliable diagnostic tool to distinguish subungual melanoma in situ from subungual benign melanocytic proliferation: melanocyte density more than 30 cells per 1 mm is highly suggestive of subungual MIS [5]. However, it is occasionally difficult to histopathologically distinguish one from another, as melanocyte density can differ significantly in subungual MIS and benign SMP depending on the skin type of the patient and the sampled area [1].

Immunohistochemistry (IHC) can be used to help in the diagnostic dilemma of benign melanocytic lesion versus melanoma in situ. However, HMB-45, highlighting the maturation of melanocyte in benign melanocytic nevus, may not be useful in the diagnosis of MIS where the dermal invasion is absent [6]. Meanwhile, p16, serving as a tumor suppressor protein in the regulation of cell cycle and senescence, has been suggested to be inactivated or lost in melanoma [7]. Nevertheless, Chu et al. [1] found that IHC staining for p16 could not distinguish subungual lentigo from subungual MIS.

Cyclin D1, a protein encoded by *CCND1* gene, is one of the key components of physiologic regulation of cell cycle. Cyclin D1 regarded as oncogenic protein to promote cell proliferation has been reported to be upregulated in various malignancies including melanoma [8]. Ramirez et al. [9] suggested higher level of cyclin D1 expression in primary melanoma compared with melanocytic nevus. Also, Preferentially expressed Antigen in Melanoma (PRAME), a tumor-associated antigen isolated by autologous T cells in a melanoma patient, was reported to be overexpressed in malignant melanomas rather than benign melanocytic lesions [10]. Lezcano et al. [11] found that over 88% of non-spindle cell primary cutaneous melanoma showed diffuse positivity for PRAME while 86% of melanocytic nevus were negative for PRAME.

However, IHC staining for cyclin D1 and PRAME has not been thoroughly investigated in melanocytic lesions of nail unit. The prevalence of subungual melanoma in Asian population is quite higher than Western population; subungual melanoma has been reported to comprise about 10–18% of cutaneous melanoma in Asian population [12, 13]. Relatively poor prognosis, distinct clinical features, and diverse genetic mutation of subungual melanoma [14–16], may indicate differences in the biology of subungual melanoma compared with other cutaneous melanoma.

Herein, we assessed the ability of promising two markers, cyclin D1 and PRAME, to distinguish subungual MIS from benign SMP, both clinically presenting as melanonychia, in a retrospective study.

## Methods

### Patient selection

This retrospective study, approved by the Institutional Review Board (IRB) of Asan Medical Center (IRB No. 2020 − 1102), included cases of benign SMP including lentigo and nevi, and subungual MIS, confirmed by biopsy at Asan Medical Center from January 2016 to December 2020. Cases of melanocytic activation without proliferation and melanoma where dermal invasion was identified were excluded.

### Variables of interest

Demographic and clinical data were investigated through reviewing electronic medical record (EMR) of Asan Medical Center, including sex, age at diagnosis, age of onset, duration of disease, location of the lesion, width of melanonychia, color of melanonychia, and presence of nail dystrophy or periungual pigmentation including Hutchinson’s sign and pseudo-Hutchinson’s sign.

Histopathologic data including melanocyte density, confluency, pagetoid melanocytosis, presence of inflammatory cell infiltration or melanophage, cytologic atypia of melanocytes, and distribution of melanin pigment were investigated through reviewing the slides by two dermatopathologists (YJK, CJJ) with agreement. Melanocyte density was measured as the number of intraepithelial melanocytes over 1 mm dermoepidermal junction of the nail matrix. As more than 30 cells per millimeter was reported to suggestive of melanoma in situ [5], we analyzed the slides according to whether melanocyte density was over 30 cells per millimeter or not. Cytologic atypia defined as nuclear enlargement, hyperchromatism, or prominent nucleoli was graded as follows: absence, mild, moderate, and severe.

### Immunohistochemical analysis

Skin tissues obtained for routine diagnostic pathologic examinations were used for IHC studies of the anti-CYCLIN D1 (1:100, Mouse monoclonal, clone SP4, catalog No.CELL MARQUE, CELL MARQUE, Rocklin,California, USA), anti-PRAME (1:1000, Rabbit polyclonal, catalog No.ab219650, ABCAM, Cambridge, UK). Formalin fixed, paraffin-embedded tissue sections were immunohistochemically stained for expression of anti-CYCLIN D1 and PRAME using a BenchMark XT automatic immunostaining device (Ventana Medical Systems, Tucson, AZ, USA) with ultraView Universal AP Red Detection Kit (Ventana Medical Systems, Tucson, AZ, USA) according to the manufacturer’s instructions.

Cyclin D1 and PRAME expression was assessed in both benign SMP and subungual MIS by counting proportion of melanocytes with nuclear positivity under 200x magnification, which was statistically analyzed to determine cutoff value regarding sensitivities and specificities, using Youden index (specificity + sensitivity − 1).

### Statistical analysis

Chi-squared test and Fisher’s exact test were used to compare the categorical variables of clinicopathologic features of subungual benign melanocytic proliferation and subungual melanoma in situ. Mann-Whitney U test was used for the continuous variables of clinicopathologic features. Sensitivities and specificities for PRAME were calculated to determine cutoff value regarding discriminatory power assessed by the area under the receiver operating characteristic curve (AUC), using MedCalc (version 20.0, MedCalc Software Ltd, Ostend, Belgium). All analyses except for were performed using SPSS (version 23.0, IBM Corp, Armonk, NY). In this study, the *p*-value of ≤ 0.05 was considered statistically significant.

## Results

A total of 14 patients and 13 patients were diagnosed with benign SMP and subungual MIS, respectively, at Asan Medical Center from January 2016 to December 2020 (Supplementary Appendix 1). Of 14 cases of benign SMP, 8 cases were diagnosed with subungual lentigo while the others were subungual melanocytic nevus. The demographic characteristics are summarized in Table 1. The cohort of benign SMP included 7 males and 7 females with a mean age of 30.71 years (range, 7–66 years). The cohort of subungual MIS included 7 males and 6 females with a mean age of 44.92 years (range, 6–74 years). The patients with subungual MIS seemed to be older than the patients with benign SMP, but the difference was not statistically significant (*p* = 0.061).

**Table 1:** Clinical features of subungual benign melanocytic proliferation and subungual melanoma in situ

Features	Subungual benign melanocytic proliferation (*n* = 14), n (%)	Subungual Melanoma in situ (*n* = 13), n (%)	*p*-value
Sex			0.842
Male	7 (50)	7 (53.8)	
Female	7 (50)	6 (46.2)	
Age, years			0.061
Range	7-66	6-74	
Mean ± SD	30.71 ± 19.83	44.92 ± 17.73	
Onset age, years			0.048^a^
Range	4-56	5-64	
Mean ± SD	25.71 ± 15.97	39.46 ± 17.16	
Prediagnosis duration, months			0.756
Range	8-240	6-180	
Mean ± SD	59.21 ± 67.27	66.62 ± 64.12	
Location			0.385
Finger	12 (85.7)	9 (69.2)	
Toe	2 (14.3)	4 (30.8)	
Width, mm			0.017^a^
Range	1.00-13.00	1.50-15.00	
Mean ± SD	3.39 ± 2.90	7.81 ± 4.87	
Background pigmentation			1.000
Yes	11 (78.6)	11 (84.6)	
No	3 (21.4)	2 (15.4)	
Nail dystrophy			0.596
Yes	0 (0.0)	2 (15.4)	
No	14 (100.0)	11 (84.6)	
Periungual pigmentation			0.568
Yes	6 (42.9)	7 (53.8)	
No	8 (57.1)	6 (46.2)	

^a^Statistically significant

### Characteristics of subungual benign melanocytic proliferation and subungual melanoma in situ

The clinical features of benign SMP and subungual MIS are summarized in Table 1. The patients with benign SMP were more likely to have melanonychia at younger age (mean ± SD, 25.71 ± 15.9) than the patients with subungual MIS (mean ± SD, 39.46 ± 17.16) (*p* = 0.048). The mean duration from the onset of the skin lesion to diagnosis of benign SMP was 59.21 months (range, 8-240 months); the mean prediagnosis duration of subungual MIS was 66.62 months (range, 6-180 months). The fingernail was involved in 12 patients with benign SMP (85.7%) and 9 patients with subungual MIS (69.2%), while toenail was involved in 2 patients with benign SMP (14.3%) and 4 patients with subungual MIS (30.8%). The width of melanonychia was significantly thinner in benign SMP (mean ± SD, 3.39 ± 2.90 mm) than subungual MIS (mean ± SD, 7.81 ± 4.87 mm) (*p* = 0.017). Colors of melanonychia striata were tan, brown, or black, with background pigmentation in 11 out of 14 patients with benign SMP (78.6%) and 11 out of 13 patients with subungual MIS (84.6%). There was no patient with benign SMP showing nail dystrophy at the involved nail. Periungual pigmentation on proximal nail fold or hyponychium suggestive of Hutchinson’s sign or pseudo-Hutchinson’s sign was observed in 6 patients with benign SMP (42.9%). When it comes to subungual MIS, nail dystrophy and periungual pigmentation was found in 2 patients (15.4%) and 7 patients (53.8%) respectively, with no significant difference compared with benign SMP.

The histopathological features of benign SMP and subungual MIS are showed in Table 2. Melanocytic density more than 30 cells per 1 mm stretch of subungual dermoepidermal junction was not statistically different between subungual MIS and benign SMP (*p* = 0.182). Focal confluency was identified significantly less in benign SMP than subungual MIS (*p* < 0.001); 5 of 14 (35.7%) cases showed focal confluency, while at least focal confluency was found in all cases of subungual MIS. The patients with benign SMP showed significantly less pagetoid melanocytosis (6 out of 14 patients) than subungual MIS (12 out of 13) (*p* = 0.006). Inflammatory cell infiltration was found in 5 of 14 patients with benign SMP (35.7%) and 5 of 13 patients with subungual MIS (38.5%), respectively. Compared with subungual MIS, cytologic atypia of benign SMP was significantly milder (*p* < 0.001); 12 out of 14 patients with benign SMP (85.7%) showed absent or mild cytologic atypia, while 10 out of 13 patients with subungual MIS (76.9%) showed moderate to severe atypia. In benign SMP cases, melanophage was found in 8 cases (57.1%), and melanin pigment limited to basilar area was identified in 4 cases (28.6%). In subungual MIS cases, melanophage was found in 9 cases (69.2%), and melanin pigment in entire epidermis was found in 9 cases (69.2%). More specifically, out of 4 benign SMP showing melanocytic density > 30 cells/mm, 2 cases were lentigines and 2 cases were nevi. All 5 benign SMP with confluency were nevi, 5 out of the 6 benign SMP cases with pagetoid melanocytosis were lentigines, and 1 case was nevus. Also, out of 8 benign SMP with atypia, 5 lentigines and 1 nevus showed mild atypia, and 2 nevi showed moderate atypia.

**Table 2:** Histopathological features of subungual benign melanocytic proliferation and subungual melanoma in situ

Features	Subungual benign melanocytic proliferation (*n *= 14), n (%)	Subungual Melanoma in situ (*n *= 13), n (%)	*p*-value
Melanocyte density			0.182
≤ 30 cells/mm	10 (71.4)	6 (46.2)	
> 30 cells/mm	4 (28.6)	7 (53.8)	
Confluency			< 0.001*
Yes	5 (35.7)	13 (100.0)	
No	9 (64.3)	0 (0.0)	
Pagetoid melanocytosis			0.006*
Yes	6 (42.9)	12 (92.3)	
No	8 (57.1)	1 (7.7)	
Inflammation			0.883
Yes	5 (35.7)	5 (38.5)	
No	9 (64.3)	8 (61.5)	
Atypia			< 0.001*
No	6 (42.9)	0 (0.0)	
Mild	6 (42.9)	3 (23.1)	
Moderate	2 (14.3)	5 (38.5)	
Severe	0 (0.0)	5 (38.5)	
Melanophage			0.516
Yes	8 (57.1)	9 (69.2)	
No	6 (42.9)	4 (30.8)	
Melanin pigment			1.000
Basilar	4 (28.6)	4 (30.8)	
Entire	10 (71.4)	9 (69.2)	
cyclin D1			1.000
Positive	4 (28.6)	3 (23.1)	
Negative	7 (71.4)	9 (66.9)	
PRAME			<0.001^a^
Positive	1 (7.1)	10 (76.9)	
Negative	13 (92.9)	3 (23.1)	

*Abbreviations: PRAME* PReferentially expressed Antigen in MElanoma

^a^Statistically significant

**Fig. 1:**
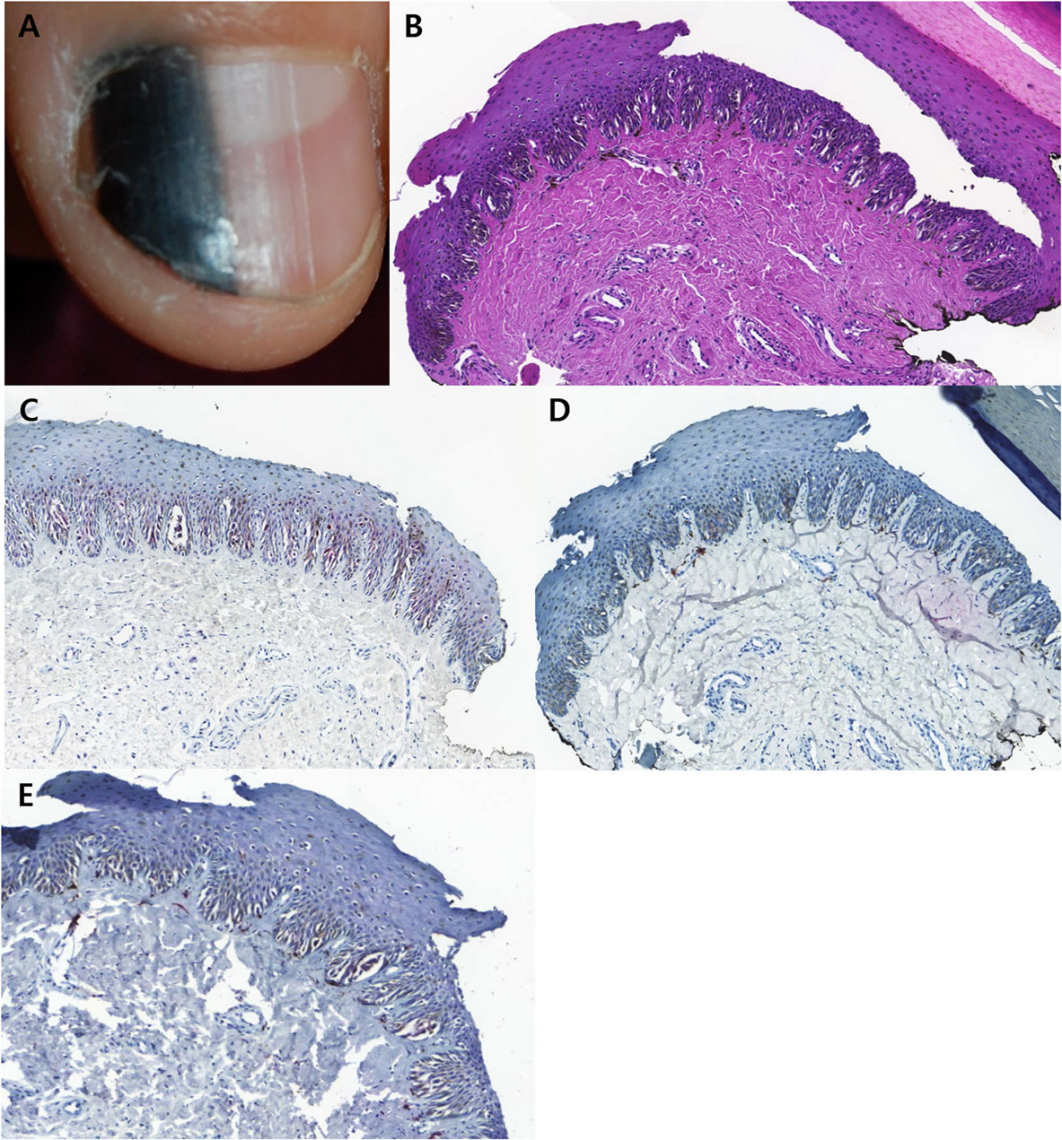
Representative clinical morphology, photomicrographs of H&E staining, and IHC staining for cyclin D1and PRAME of selected subungual benign melanocytic proliferation. One 7-year-old patient presenting with (**A**) 3.5 mm-wide melanonychia showed (**B**) melanocyte proliferation showing mild atypia without confluency or pagetoid spread (200x magnification, H&E). (**C**) While cyclin D1 IHC showed over 70% nuclear immunostaining (200x magnification), (**D**) PRAME IHC exhibited total negativity (200x magnification), (**E**) Sox-10 IHC (200x magnification)

**Fig. 2:**
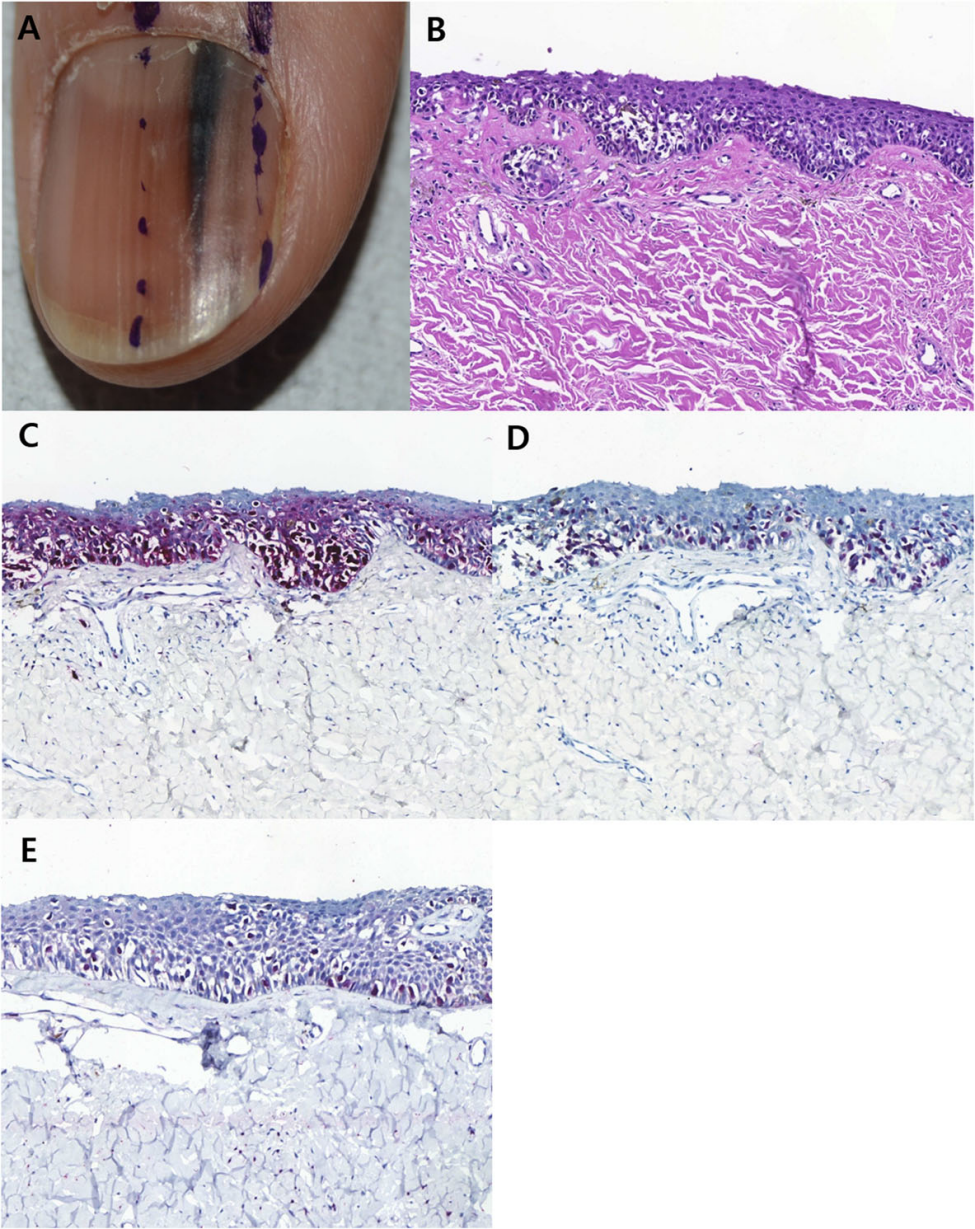
Representative clinical morphology, photomicrographs of H&E staining, and IHC staining for cyclin D1 and PRAME of selected subunugal melanoma in situ. One 44-year-old patient (**A**) 2 mm-wide melanonychia showed (**B**) atypical melanocyte proliferation with confluency and pagetoid spread (200x magnification, H&E). Both (**C**) cyclin D1 and (**D**) PRAME IHC showed over 90% nuclear immunostaining (200x magnification, respectively), (**E**) Sox-10 IHC (200x magnification)

The results of IHC staining are summarized in Table 3. When it comes to benign SMP, four cases (28.6%) showed ≤ 20% nuclear immunostaining for cyclin D1, while 11 cases (71.4%) exhibited > 70% nuclear immunostaining for cyclin D1. 13 cases (92.9%) exhibited totally negative staining for PRAME, while 1 case (7.1%) showed ≤ 20% nuclear immunostaining for PRAME (Fig. 1). In terms of subungual MIS, eight cases (61.5%) showed ≤ 20% nuclear immunostaining for cyclin D1, while 5 cases (38.5%) exhibited > 60% nuclear immunostaining for cyclin D1. 3 cases (23.1%) were totally negative for PRAME, while 10 cases (76.9%) exhibited > 50% nuclear immunostaining for PRAME (Fig. 2).

**Table 3:** Immunohistochemistry staining of subungual benign melanocytic proliferation and subungual melanoma in situ

	Subungual benign melanocytic proliferation (*n* = 14), n (%)	Subungual Melanoma in situ (*n* = 13), n (%)
cyclin D1 nuclear immunostaining		
0%	2 (14.3)	2 (15.4)
> 0–10%	0 (0.0)	2 (15.4)
> 10–20%	2 (14.3)	4 (30.8)
> 20–30%	0 (0.0)	0 (0.0)
> 30–40%	0 (0.0)	0 (0.0)
> 40–50%	0 (0.0)	0 (0.0)
> 50–60%	0 (0.0)	0 (0.0)
> 60–70%	0 (0.0)	1 (7.7)
> 70–80%	3 (21.4)	0 (0.0)
> 80–90%	3 (21.4)	1 (7.7)
> 90–100%	4 (28.6)	3 (23.1)
PRAME nuclear immunostaining		
0%	13 (92.9)	3 (23.1)
> 0–10%	0 (0.0)	0 (0.0)
> 10–20%	1 (7.1)	0 (0.0)
> 20–30%	0 (0.0)	0 (0.0)
> 30–40%	0 (0.0)	0 (0.0)
> 40–50%	0 (0.0)	0 (0.0)
> 50–60%	0 (0.0)	3 (23.1)
> 60–70%	0 (0.0)	0 (0.0)
> 70–80%	0 (0.0)	1 (7.7)
> 80–90%	0 (0.0)	2 (15.4)
> 90–100%	0 (0.0)	4 (30.8)

*Abbreviations:* *PRAME* PReferentially expressed Antigen in MElanoma

### Sensitivity and specificity of cyclin D1 and PRAME

**Fig. 3:**
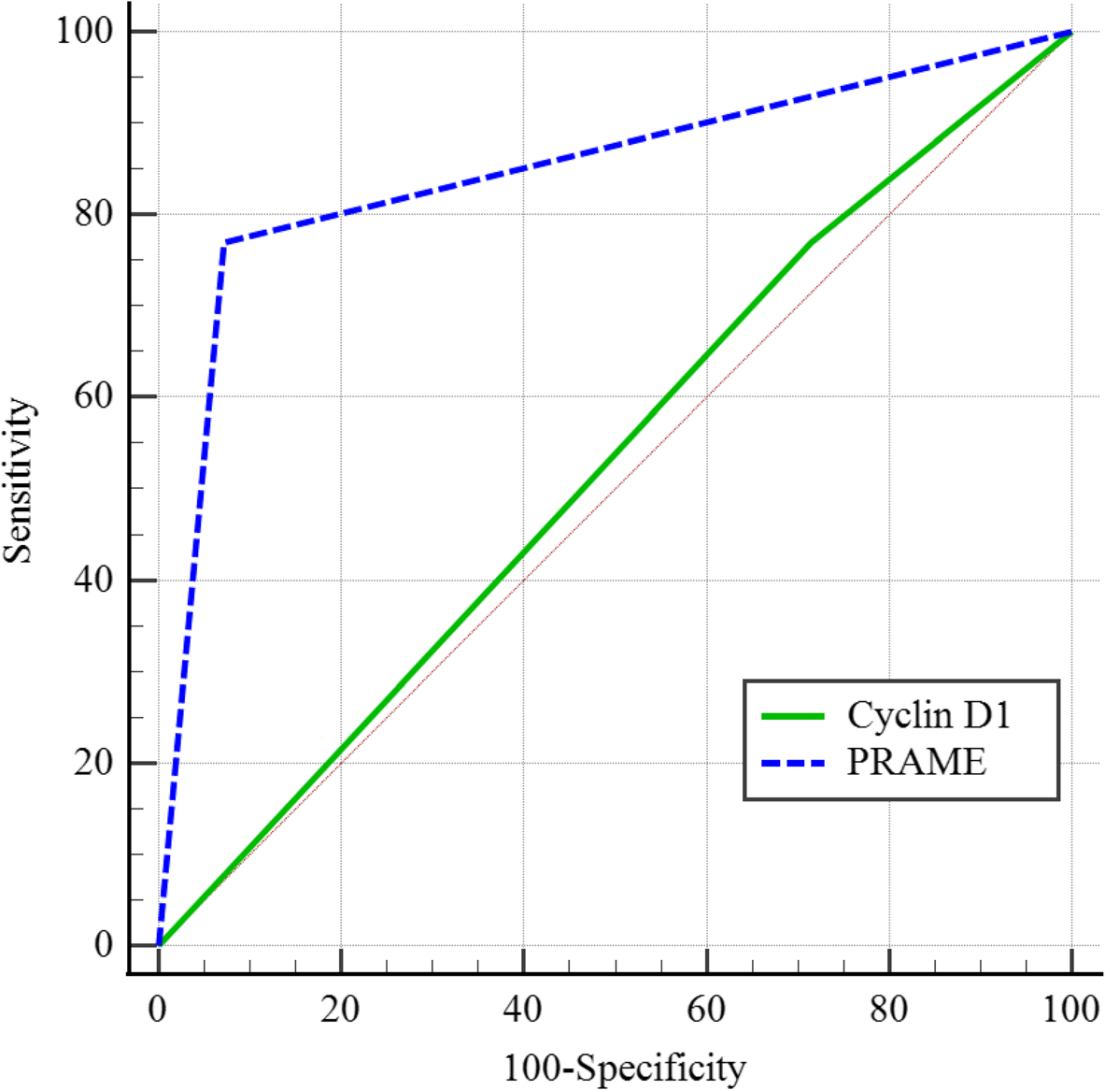
Receiver Operating Characteristic (ROC) curve for discriminating subungual MIS from benign SMP based on the expression levels of Cyclin D1 and PRAME. Using the cutoff of 92.5%, with > 92.5% nuclear immunostaining for cyclin D1 as a positive test for subungual MIS, cyclin D1 showed poor overall discrimination between benign SMP and subungual MIS (AUC = 0.527, 95% CI = 0.328–0.721). Using the cutoff of 10%, with > 10% nuclear immunostaining for PRAME as a positive test for subungual MIS, PRAME exhibited good overall discrimination between benign SMP and subungual MIS (AUC = 0.849, 95% CI = 0.659–0.957)

Using the cutoff of 92.5%, with > 92.5% nuclear immunostaining for cyclin D1 as a positive test for subungual MIS, the sensitivity, specificity, positive predictive value (PPV), and negative predictive value (NPV) of cyclin D1 to distinguish subungual MIS from benign SMP were 23.1%, 85.7%, 21.6%, and 91.7%, respectively. At this cutoff, cyclin D1 showed poor overall discrimination between benign SMP and subungual MIS (AUC = 0.527, 95% CI = 0.328–0.721) (Fig. 3). There was no significant differences in the positivity of cyclin D1 with cutoff of 92.5% between two groups (*p* = 1.000) (Table 2).

Using the cutoff of 10%, with > 10% nuclear immunostaining for PRAME as a positive test for subungual MIS, the sensitivity, specificity, PPV, and NPV of PRAME for distinguishing subungual MIS from benign SMP were 76.9%, 92.9%, 71.6%, and 99.8%, respectively. At this cutoff, PRAME exhibited good overall discrimination between benign SMP and subungual MIS (AUC = 0.849, 95% CI = 0.659–0.957) (Fig. 3), which distinguished two groups significantly better than cyclin D1 (*p* = 0.013). PRAME was significantly more expressed in subungual MIS than benign SMP (*p* < 0.001) (Table 2).

## Discussion

Distinguishing subungual melanoma from non-nail apparatus acral melanoma is clinically important due to its distinct clinical courses including high recurrence rate and short progression-free survival [14, 15]. Also, it has been reported that subungual melanoma harbors more distinct genomic alterations including *CARD11*, *ARID2*, *ARID1A*, *ARID1B*, *PTPRB*, and *PTPRK* genes, compared with acral melanoma [17].

Cyclin D1 in coordination with their catalytic partners CDK4 and CDK6, contributes to promoting cell cycle progression through transition from G1 to S phase [18]. Cyclin D1 upregulation with amplification of *CCND1* has been documented in various malignancies including breast, lung, colon, and oral cancers [19]. Meanwhile, the expression rate of cyclin D1 in cutaneous melanoma compared with benign melanocytic nevus has been discordantly reported [9, 20, 21]. When it comes to acral melanoma, cyclin D1 has been reported to be overexpressed, resulting in constitutively activated MAPK signaling pathway without *NRAS* or *BRAF* mutations [22–24]. In this regard, we had expected cyclin D1 IHC staining would be an effective discriminator of benign SMP from subungual MIS.

However, cyclin D1 IHC staining seemed not to be reliable in distinguishing benign SMP from subungual MIS, in this study. Positive nuclear immunostaining for cyclin D1 was found in 4 out of 14 (28.6%) patients with benign SMP and 3 out of 13 (23.1%) patients with subungual MIS using cutoff of 92.5% which was determined regarding sensitivities and specificities using Youden index, with poor sensitivity of 23.1%. The lack of diagnostic value of cyclin D1 overexpression in these lesions may be due to the differences in the type of antibody used, the positive cell count system, and cutoff point for positivity [8]. Also, it is important to note that cyclin D1 overexpression does not always represent amplification of *CCND1*; loss of cyclin D1 IHC staining does not always represent loss of function of *CCND1*, vice versa. Epigenetic regulation including DNA methylation at cytosine and histone acetylation can cause alteration in mRNA and protein expression [25]. Therefore, further genetic tests including fluorescent *in situ* hybridization (FISH) for *CCND1* may be needed for determining true *CCND1* amplification to discriminate between benign SMP and subungual MIS [22].

PRAME gene is a member of cancer testis antigen (CTA) gene family encoding a membrane-bound protein recognized by T lymphocytes, causing autologous cytotoxic T cell-mediated immune response [26]. Except for some distinct tissues including testis, ovary, placenta, adrenals, and endometrium, PRAME is not detected in healthy human tissues [10]. Overexpression of PRAME was found to inhibit retinoic acid (RA) mediated cell differentiation, cell growth arrest, and apoptosis, contributing to tumorigenesis via inhibiting RA receptor signaling [27]. PRAME has been reported to be overexpressed in a variety of malignancies including malignant melanoma, showing an utility in distinguishing between benign and malignant lesions [28]. Although little has been studied about the diagnostic utility of PRAME in subungual melanocytic lesions, a recently published paper has validated the usefulness of PRAME expression in differentiating between melanoma and other nail unit melanocytic lesions [29]. In the paper, subungual melanomas were all positive in PRAME IHC, and benign melanocytic lesions of nail unit were all negative. However, out of the 25 melanoma cases, 20 cases were invasive melanoma, while only 5 cases were MIS. Our paper provides additional insight from current literature focusing on MIS, which is more difficult to distinguish from benign lesions.

In this study, we demonstrated that PRAME IHC staining was a relia4ble discriminator of benign SMP from subungual MIS. Positive nuclear immunostaining for PRAME was found in 1 out of 14 (7.1%) patients with benign SMP and 10 out of 13 (76.9%) patients with subungual MIS using cutoff of 10%, showing modest sensitivity of 76.9% and good specificity of 92.9%. Cutoff (10%) for positivity of PRAME in this study is quite different from previously reported cutoff values ranging from 50 to 75% [7, 10, 28]. Differences in cutoff values among studies may be due to divergences in IHC staining methodology and inter-observer variability in IHC assessment [28]. Also, the type of melanocytic lesions included in the studies might affect the differences in cutoff value for positivity of PRAME. Unlike previous studies, we assessed the expression of PRAME using only subungual melanocytic lesions, known to have different genomic alterations with lower mutation burden compared with other cutaneous melanomas [23, 30]. Also, small sample size of this study might have influenced the difference in cutoff value. Therefore, additional studies of PRAME IHC in a large cohort of melanocytic lesions with different subtypes are needed to determine whether cutoff value for PRAME positivity should differ according to the subtype of melanocytic lesions, or not.

Compared with cytogenetic studies including FISH, IHC has several advantages including more rapid turnaround time, lower cost, and higher accessibility. However, PRAME IHC can exhibit false positive and false negative results, confusing the correct diagnosis. Lezcano et al. [11] reported that diffuse nuclear immunoreactivity for PRAME was found in 13.6% of cutaneous melanocytic nevus. Also, Shyam et al. [28] demonstrated focal immunopositivity of PRAME from 5 to 10% in atypical but benign non-spitzoid melanocytic proliferations. In this study, 1 out of 14 (7.1%) patients with benign SMP exhibited PRAME nuclear immunostaining ranging from 10 to 20%. The significance of focal PRAME positivity in benign melanocytic lesions is still unclear, which necessitate further studies to determine the potential risk of malignant transformation related to focal PRAME positivity. Additional cytogenetic and molecular tests can help avoiding overdiagnosis in the cases with focal PRAME positivity [10].

Also, it has been reported that primary cutaneous melanoma could be completely negative for PRAME immunostaining [10]. In this study, 3 out of 13 (23.1%) patients showed completely negative PRAME IHC. Histopathological analysis revealed there was no melanophage adjacent to cutaneous melanoma in these patients. Melanophage found in cutaneous melanoma is indicative of immune responses, predicting a relatively good prognosis for patients, possibly through tumor regression due to phagocytosis of melanoma cells [31]. As PRAME gene encodes a membrane-bound protein recognized by T lymphocytes causing autologous cytotoxic T cell-mediated immune response [26], it is possible that lack of PRAME expression may lead to decreased immune response and reduced melanophages. While PRAME was found to be related with metastasis in uveal melanoma [32], little has been reported about the prognostic value of PRAME expression in cutaneous melanoma. Further studies are needed to determine the prognostic value of PRAME expression in cutaneous melanoma, regarding the role of PRAME in the relationship between immune response and oncogenesis.

It is important to keep in mind that IHC should be used in conjunction with clinical findings and histological analysis to make a final diagnosis. Clinically, we found that the patients with subungual MIS tended to be older and have wider lesions than the patients with benign SMP, with statistical significance. We also demonstrated that the patients with subungual MIS showed significantly more confluency, pagetoid melanocytosis, and severe atypia than the patients with benign SMP. Combining these clinical and histopathological features with PRAME IHC may significantly increase diagnostic power to distinguish benign SMP from subungual MIS.

This study has several limitations. First, this study was a retrospective study with relatively small sample size. Small sample size limited the usage of multivariate logistic regression analysis to develop a scoring system for the diagnosis of subungual melanoma in situ using PRAME expression. Second, this study was a single-center study confined to Korean patients. As the difference in the prevalence rate of subungual melanoma between the Asian group and other races including Western group is prominent enough to suppose difference in the biology of subungual melanoma in each group. Therefore, cyclin D1 and PRAME expression in melanocytic lesions of nail unit should be studied in other races including Western group. Also, this study evaluated no cytogenetic or molecular studies to compare with PRAME IHC in the diagnosis of subungual MIS.

## Conclusions

This study suggests that PRAME IHC staining as a reliable discriminator in distinguishing subungual MIS from benign SMP. We also demonstrated PRAME expression should be interpreted in the context of histopathologic features. Further studies using larger cohort and ancillary studies including cytogenetic studies are needed to confirm the diagnostic utility of cyclin D1 and PRAME expression in the diagnosis of subungual MIS.

## Supplementary Information

**Additional file 1: Supplementary Appendix 1.** Clinical and histopathological data for all cases included.

## Data Availability

The data that support the findings of this study are available from the corresponding author upon reasonable request.

## Abbreviations

IHC: Immunohistochemistry
MIS: Melanoma in situ
SMP: Subungual melanocytic proliferation
PRAME: Preferentially expressed Antigen in Melanoma
PPV: Positive predictive value
NPV: Negative predictive value
CTA: Cancer testis antigen
RA: Retinoic acid
